# Impacts of Global Climate Change on Potential Habitat Distribution and Range Shifts of Semi-Subterranean Rodents

**DOI:** 10.3390/ani16142245

**Published:** 2026-07-20

**Authors:** Die Chen, Rong Zhang, Helong Yang, Suwen Yang, Wenshan Chen, Mengyue Wang, Jiuqi Zhao

**Affiliations:** 1 College of Grassland Science, Xinjiang Agricultural University, Urumqi 830052, China; 18085849247@163.com (D.C.); yangsuwen2014@126.com (S.Y.); 15863671865@163.com (W.C.); 15035964969@163.com (M.W.); xndzjq@163.com (J.Z.); 2 Xinjiang Key Laboratory of Grassland Resources and Ecology, Urumqi 830052, China; 3 Key Laboratory of Grassland Resources and Ecology of Western Arid Region, Ministry of Education, Urumqi 830052, China

**Keywords:** mole vole (*Ellobius tancrei*), biomod2, suitable habitat, centroid migration

## Abstract

As integral components of ecosystems, rodents exhibit high sensitivity to environmental fluctuations. As a typical semi-subterranean rodent, the burrowing and foraging behaviors of the mole vole (*Ellobius tancrei*) profoundly impact local microhabitat structure and vegetation succession. Given its extensive distribution range, investigating the geographical distribution of the mole vole under climate change is critical for understanding its population dynamics and spatial patterns. To explore the impacts of climate change on the potential distribution of this species, we utilized an ensemble model based on 395 occurrence records and 38 environmental variables. We predicted its potential geographical distribution under current (1970–2000) and three future climate scenarios (SSP126, SSP245, and SSP585) across three future periods (2041–2060, 2061–2080, and 2081–2100) and further analyzed the variation trends and centroid migration directions of its global potential distribution. The results revealed the following: (1) Elevation (Dem) and temperature annual range (Bio7) were the dominant environmental factors driving the potential distribution of the mole vole, followed by precipitation of the warmest quarter (Bio18), mean diurnal range (Bio2), annual mean temperature (Bio1), base saturation (BSAT), precipitation seasonality (Bio15), calcium carbonate content (TCARBON_EQ), and available water capacity (AWC). (2) Under current climate conditions, the highly suitable habitats were mainly concentrated in Eurasia, particularly in Turkey, Kyrgyzstan, China, Mongolia, and Russia. (3) Under future climate scenarios, suitable habitats showed simultaneous expansion and contraction, with expansion consistently exceeding contraction across all scenarios and periods, especially under SSP585 in the late 21st century. (4) Relative to the current period, the suitable-habitat centroid shifted northeastward under SSP126, but northwestward under SSP245 and SSP585. This study reveals the dynamic spatial shifts of the mole vole driven by climate change, not only providing crucial scientific support for early spatial warning and precise prevention of agricultural and pastoral rodent damage, but also laying a theoretical foundation for understanding the ecological adaptation mechanisms of subterranean rodents and mitigating potential cross-regional dispersal risks.

## Simple Summary

Global climate change threatens both environmental stability and human health. The mole vole, a semi-subterranean rodent living across diverse landscapes, causes severe damage to agriculture. More importantly, it carries a highly lethal disease caused by parasitic worms that can spread to humans. This study aims to predict how changing weather patterns will force these rodents to shift their living areas. Using computer models based on current and future climate data, our results show their suitable habitats will actually expand. Specifically, their living areas will likely shift towards farmlands. As climate change disrupts natural food sources, human-managed farmlands offer stable and abundant food supplies, ensuring their survival. These findings are highly valuable. They not only offer early warnings to protect agriculture from rodent damage, but also provide crucial information for public health officials to prevent the cross-regional spread of a deadly animal-to-human disease. Ultimately, tracking these spatial shifts helps us to manage the deep connection between wildlife adaptation, food security, and public safety in a warming world.

## 1. Introduction

The geographical distribution of a species is the outcome of its adaptive selection of different habitats [1]. Climate represents one of the most critical determinants of species distribution, and climate change exerts profound impacts on global biodiversity [2,3], ecosystem functioning [4], human health [5], and species’ geographical distributions [6,7]. The mechanisms through which climate change affects both aboveground and subterranean rodents are multifaceted. Rising temperatures induce the melting of glaciers and the degradation of permafrost in high-altitude and high-latitude regions, thereby altering rodent habitats. Concurrently, variations in temperature and precipitation influence plant phenology, subsequently affecting rodents’ food resources, thus driving shifts in their distribution ranges [8] and even causing centroid migration of their suitable habitats. Furthermore, elevated temperatures can increase rodents’ metabolic rates and energy expenditure, ultimately impacting their reproductive physiology. Drought leads to soil compaction and reduced aeration; in severe cases, it causes burrows to desiccate and collapse, creating detrimental conditions for subterranean rodents. Conversely, excessive precipitation can inundate subterranean burrow systems, posing a severe threat to their survival [9,10].

*Ellobius tancrei* is widely distributed from eastern Turkmenistan and Uzbekistan through eastern Kazakhstan to Mongolia and adjacent regions of China, including northwestern Xinjiang, Inner Mongolia, northern Shaanxi, northern Gansu, and Ningxia [11]. Regional surveys have also recorded this species in the Altay border area of northwestern China [12], and it is considered a common rodent species in northwestern China [13]. Its vertical distribution ranges from piedmont deserts to subalpine meadows, with the highest population densities occurring within mountain forests, meadow steppes, and mountain steppes [14]. The mole vole primarily inhabits subterranean environments and occasionally engages in aboveground activities [15], characterizing it as a typical semi-subterranean rodent.

Rodents play vital ecological roles in grassland ecosystems [16]. According to the intermediate disturbance hypothesis, local species diversity peaks under moderate levels of ecosystem disturbance [17]. Under such conditions, the excavation behaviors of rodents can ameliorate soil conditions, and their mound-building activities create heterogeneous microhabitats, thereby facilitating the maintenance of plant diversity [18,19].

Conversely, mole voles predominantly feed on plant roots and tubers; their excessive foraging and excavating behaviors lead to root damage and a reduction in vegetation coverage, respectively. This causes severe degradation of pastures, declines in forage yield, and the formation of uneven micro-topography [20]. Such disruptions further exacerbate grassland degradation, occasionally resulting in the emergence of “rodent-degraded wastelands.” This process creates a vicious cycle by transforming the grassland into a habitat even more conducive to the colonization and reproduction of pest rodents [21]. Furthermore, the mole vole serves as one of the intermediate hosts for alveolar echinococcosis (AE) [22], a highly lethal zoonotic helminthic disease that poses a severe threat to human health [23].

To date, domestic and international studies on the mole vole (*E. tancrei*) have predominantly focused on its behavioral patterns [24,25], impacts on pasture degradation [20], population outbreaks [26], prevention and control strategies [27,28], age determination [29], and regional spatial distribution [30]. However, our systematic understanding of its geographical distribution at the global scale remains notably limited. Therefore, investigating the spatial dynamics of the mole vole under climate change scenarios is crucial for comprehending its population dynamics and macro-ecological patterns.

Currently, in studies exploring the impacts of climate change on species distributions, the primary species distribution models (SDMs) employed include Random Forest (RF) [31], Maximum Entropy (Maxent) [1,32], Classification and Regression Trees (CARTs) [33], Ecological Niche Factor Analysis (ENFA) [34], and Generalized Linear Models (GLMs) [35]. Due to inherent differences in algorithms, theoretical foundations, and assumptions, each model possesses unique advantages and limitations [36]. Furthermore, the predictive performance of individual models can become unstable when input data vary [37].

To enhance predictive accuracy and address the uncertainties and potential unreliability associated with individual models, an increasing number of researchers are focusing on ensemble modeling. By comprehensively analyzing the commonalities, differences, and uncertainties across the outputs of all constituent models, ensemble approaches can effectively mitigate the uncertainties arising from diverse modeling techniques and non-independent evaluation samples [38].

In this study, based on field surveys and the compilation of global occurrence records of the mole vole (*E. tancrei*), combined with biological, climatic, and topographic environmental factors, we utilized an ensemble model to predict the animal’s global potential geographical distribution. Furthermore, we analyzed the dominant environmental factors driving its distribution and elucidated the variation trends and centroid migration directions of its suitable habitats globally under various future climate scenarios.

## 2. Materials and Methods

### 2.1. Acquisition and Processing of E. tancrei Occurrence Data

The mole vole occurrence data used herein were obtained using the following three approaches: (1) From 2023 to 2025, comprehensive field surveys were conducted across the Xinjiang Uygur Autonomous Region, China (encompassing Tacheng Prefecture, Changji Hui Autonomous Prefecture, Altay Prefecture, Ili Kazakh Autonomous Prefecture, Hami, Bayingolin Mongol Autonomous Prefecture, Kizilsu Kirghiz Autonomous Prefecture, Turpan, Karamay, Bortala Mongol Autonomous Prefecture, and Urumqi), yielding a total of 130 in situ occurrence records. (2) A total of 265 occurrence records (spanning from 1991 to 2026) were retrieved from the Global Biodiversity Information Facility (GBIF, https://www.gbif.org/citation-guidelines (accessed on 29 June 2026)), comprising 27 human observations, and 238 preserved specimens.

**Figure 1:**
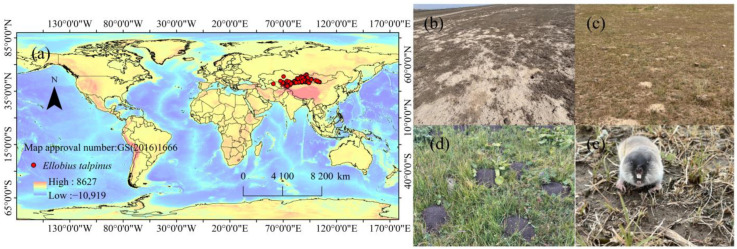
(**a**) Occurrence points of *E. tancrei* populations worldwide (red circles denote the points where *E. tancrei* is present); (**b**) temperate desert; (**c**) temperate desert steppe; (**d**) temperate meadow steppe; and (**e**) *E. tancrei*.

Based on the aforementioned sources, an initial dataset consisting of 395 distribution records was compiled. Records lacking precise spatial coordinates, duplicates, and records overtly deviating from the known ecological range of the species were rigorously excluded. Furthermore, because spatial clustering of occurrence records can exacerbate model overfitting, spatial rarefaction was conducted to mitigate clustering effects. Specifically, we only retained a single occurrence point within each 5 km × 5 km grid cell, utilizing the spThin package (version 0.2.0.) in R to eliminate spatial redundancy. Ultimately, 136 valid and independent occurrence records were retained for subsequent modeling (Figure 1).

### 2.2. Acquisition and Processing of Environmental Variables

To identify the key drivers of the mole vole’s geographical distribution, 38 environmental variables were initially selected, including 19 bioclimatic variables, 16 soil variables, and 3 topographic variables. Current and future bioclimatic data, alongside the Digital Elevation Model (DEM), were obtained from WorldClim (https://www.worldclim.org (accessed on 20 December 2024)). For future projections, the BCC-CSM2-MR global climate model was adopted for the periods 2041–2060, 2061–2080, and 2081–2100 under three Shared Socioeconomic Pathways (SSP126, SSP245, and SSP585). Among these, SSP1-2.6 represents a sustainable development scenario with low greenhouse gas emissions; SSP2-4.5 describes a ‘middle-of-the-road’ scenario with intermediate emissions, where historical patterns of development are maintained; and SSP5-8.5 illustrates a fossil-fueled development scenario with very high greenhouse gas emissions. Soil data (Version 2.0) were downloaded from the Harmonized World Soil Database (HWSD) via the FAO portal (https://gaez.fao.org/pages/hwsd (accessed on 21 December 2024)).

We extracted 16 soil factors for two soil depth intervals (0–20 cm and 20–40 cm) using the “Lookup” function in the “Reclass” toolset of ArcGIS 10.8.1. These factors included available water capacity (AWC), depth of the top of the layer (TOPDEP), sand content, clay content, SOTER soil texture classification, pH, total nitrogen (N), carbon-to-nitrogen ratio (C/N), cation exchange capacity (CEC_SOIL and CEC_CLAY) of the soil and of the clay fraction, base saturation, exchangeable sodium percentage (ESP), calcium carbonate content (CaCO_3_), gypsum content (GYPSUM), and electrical conductivity (EC). Specifically, TOPDEP was processed using R4.5.2 software to represent the 0–40 cm layer, while the remaining soil factors were averaged or summed to the 0–40 cm depth using the Raster Calculator in ArcGIS. Slope and aspect were derived from the elevation data using the “Surface Analysis” tool in ArcGIS. All environmental variables were resampled to a unified resolution of 2.5 arcminutes (~5 km) using the “Resample” tool in ArcGIS 10.8.1. Due to the lack of future projections for topography and soil, these variables were assumed to remain constant over time.

To prevent model overfitting and multicollinearity, we employed a multi-step screening process. First, we ran the MaxEnt model 10 times to calculate the average contribution rate of each variable. Second, we calculated Pearson correlations between variables; for pairs with a correlation coefficient |r| ≥ 0.8, we retained the variable that made the greater contribution to the initial model. Finally, we removed redundant variables with a Variance Inflation Factor (VIF) ≥ 10. Ultimately, 15 key environmental variables were retained for the final modeling (Table 1).

### 2.3. Model Selection and Evaluation

Ensemble species distribution modeling was performed using the biomod2 package in R (version 4.4.2). To explore the complex relationships between the distribution of the mole vole and environmental factors, twelve distinct modeling algorithms were employed: Artificial Neural Networks (ANNs), Classification Tree Analysis (CTA), Flexible Discriminant Analysis (FDA), Generalized Additive Models (GAMs), Gradient-Boosting Machines (GBMs), Generalized Linear Models (GLMs), Multivariate Adaptive Regression Splines (MARS), Maximum Entropy (MAXENT.Phi), Random Forest (RF), Random Forest-dismo (RFd), Surface Range Envelope (SRE), and Extreme Gradient Boosting (XGBOOST).

Data were formatted using the BIOMOD_FormatingData function. Following the parameter configurations, three independent sets of pseudo-absence (PA) data were randomly generated within the study area, with each set containing 1000 points, totaling 3000 PA points. For model calibration and validation, the formatted dataset was split, with 75% of the occurrence records randomly selected as the training set and the remaining 25% reserved as the testing set. Model predictive performance was evaluated using two complementary metrics: the Area Under the Receiver Operating Characteristic Curve (AUC) and the True Skill Statistic (TSS). Furthermore, the relative importance of environmental variables was quantified through three permutation runs to identify the dominant drivers of the species’ distribution.

According to the criteria established by Allouche et al. [39], AUC values range from 0.5 to 1.0, with values between 0.9 and 1.0 indicating excellent performance. TSS values measure the balance between sensitivity and specificity, with ranges of [0.55, 0.7), [0.7, 0.85), and [0.85, 1.0] representing fair, good, and excellent predictive accuracy, respectively. In this study, only individual models meeting the thresholds of a TSS ≥ 0.7 and an AUC ≥ 0.8 were selected for the final ensemble. The ensemble model was constructed by using the weighted average method to integrate the outputs of the selected individual models, generating potential geographical distribution maps under both current and future climate scenarios.

### 2.4. Classification of Suitable Habitats and Centroid Shifts

Based on the output of the ensemble model, the threshold was determined using the maximum training sensitivity plus specificity (MaxSSS) rule, resulting in a cutoff of 0.288. The probability of occurrence (*p*) was classified into four grades: unsuitable habitats (*p* ≤ 0.288), marginally suitable habitats (0.288 ≤ *p* < 0.577), moderately suitable habitats (0.577 ≤ *p* < 0.865), and highly suitable habitats (*p* ≥ 0.865). Using the “Reclassify” tool in ArcGIS, the continuous suitability maps for current and future scenarios were reclassified into these four categories.

To quantify spatial changes in distribution, we calculated the areas of range contraction, stability, and expansion for *E. tancrei* under each future scenario relative to the current distribution. This analysis was performed using the terra package (version 1.8.70) in the R software (version 4.5.2) environment.

The resulting raster layers were imported into ArcGIS to analyze centroid shifts. First, the raster data were converted into vector polygons using the “Raster to Polygon” tool. The geometric centroid of the suitable habitat for each period and scenario was then calculated using the “Mean Center” tool. Finally, the migration trajectories were reconstructed, and the direction and distance of centroid shifts between adjacent time periods were quantified using the “Points to Line” and “Split” tools.

## 3. Results and Analysis

### 3.1. Model Performance Evaluation

Among the 12 initial modeling algorithms, the Artificial Neural Network (ANN) failed to converge when trained on the full dataset, producing only a single invariant prediction value; consequently, it was excluded from further analysis. The remaining 11 models were successfully run. The Random Forest (RF) model exhibited the best performance, achieving perfect scores for both TSS and AUC (1.0). In contrast, the Surface Range Envelope (SRE) model yielded the lowest accuracy (TSS = 0.589, AUC = 0.794) and failed to meet the selection criteria (TSS ≥ 0.7, AUC ≥ 0.8), leading to its exclusion. The remaining models all met the screening thresholds.

**Figure 2:**
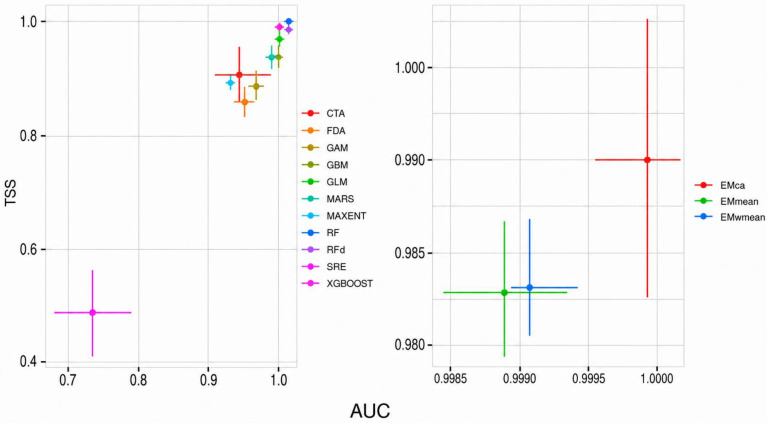
Evaluation of predictive performance for individual models under current climatic conditions. The coordinate axes represent the True Skill Statistic (TSS) and the Area Under the Receiver Operating Characteristic Curve (AUC), respectively. An AUC value closer to 1 indicates higher predictive accuracy and credibility, while a TSS value closer to 1 signifies a superior balance between model sensitivity and specificity. Abbreviations: ANN, Artificial Neural Network; CTA, Classification Tree Analysis; FDA, Flexible Discriminant Analysis; GAM, Generalized Additive Model; GBM, Gradient Boosting Machine; GLM, Generalized Linear Model; MARS, Multivariate Adaptive Regression Splines; MAXENT, Maximum Entropy; RF, Random Forest; RFd, Random Forest-dismo; SRE, Surface Range Envelope; XGBOOST, Extreme Gradient Boosting.

Ultimately, ten algorithms—CTA, FDA, the GAM, GBM, the GLM, MARS, MaxEnt, RF, RFd, and XGBoost—were selected to construct the ensemble model. The final ensemble model achieved a TSS of 0.982 and an AUC of 0.999 (Figure 2, Table 2). These metrics indicate excellent predictive performance and high reliability, confirming the model’s suitability for projecting the potential suitable habitat of *E. tancrei*.

### 3.2. Potential Global Potential Distribution of E. tancrei Under Current Climate Conditions

**Figure 3:**
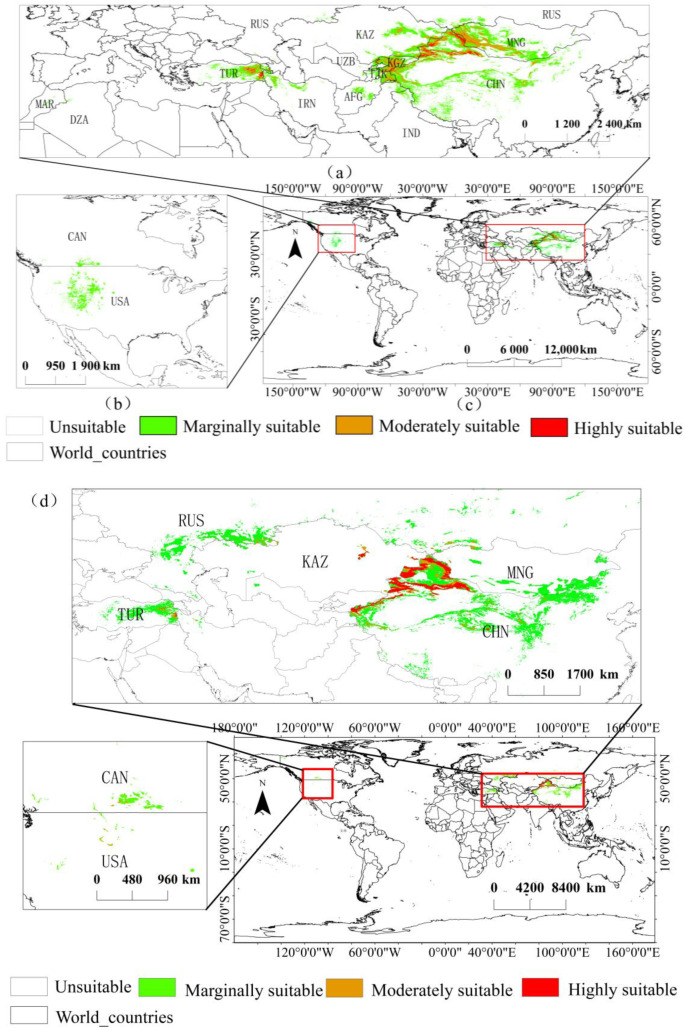
Climatic suitability zoning for *E. tancrei* under current climate conditions (1970–2000). (**a**,**b**,**d**) Labels in the figure represent abbreviations of country names. Codes in the figure represent ISO country abbreviations: MAR, Morocco; DZA, Algeria; TUR, Turkey; KAZ, Kazakhstan; UZB, Uzbekistan; IRN, Iran; KGZ, Kyrgyzstan; TJK, Tajikistan; AFG, Afghanistan; RUS, Russia; CHN, China; CAN, Canada; USA, the United States; IND, India; RUS, Russian Federation; MNG, Mongolia. (**a**–**c**) Climate suitability zoning maps of *E. tancrei* based on the multi-source full dataset model. (**d**) Climate suitability zoning map of *E. tancrei* based on the pure field dataset model.

An ensemble model was reconstructed and run using only 127 high-precision field survey records for sensitivity comparison analysis (Figure 3d). The sensitivity analysis showed that the model based purely on field data achieved high simulation accuracy (AUC = 0.999, TSS = 0.995), but its spatial projection of climatic suitability exhibited obvious spatial overfitting and niche truncation. Since field sampling points were geographically concentrated in Xinjiang, China, the model only fitted the local climatic characteristics and failed to reflect the environmental tolerance range of the species in other distribution regions outside Xinjiang. Compared with the predictions of the multi-source full dataset model, the highly suitable habitat areas predicted by the pure field-data model contracted significantly outside Xinjiang, showing a clear deviation from the actual macro-geographical distribution pattern of *E. tancrei* (Figure 3a,d).

Regional comparison indicated that the two models exhibited a high consistency in the spatial pattern of suitable habitats within Xinjiang. The ensemble model based on the multi-source full dataset also maintained high simulation accuracy (AUC = 0.999, TSS = 0.982) (Table S1). Its predicted suitable habitats at the global scale presented a continuous spatial pattern, which was more consistent with the actual geographical distribution characteristics of the species.

According to the predictive results yielded by the ensemble model under current climatic conditions (Figure 3a,b), the highly suitable habitats for the mole vole are primarily located in TUR, KGZ, CHN, MNG, and RUS. The moderately suitable habitats are mainly distributed across TUR, IRN, KGZ, TJK, RUS, CHN, MNG, and KAZ. The marginally suitable habitats are predominantly found in TUR, IRN, RUS, KAZ, KGZ, TJK, CHN, AFG, CAN, USA, MNG, and other countries. The total areas of the marginally, moderately, and highly suitable habitats are 2.5 × 10^6^ km^2^, 0.7 × 10^6^ km^2^, and 8 × 10^4^ km^2^, respectively.

The results yielded by the ensemble model indicate that the dominant environmental factors influencing the potential geographical distribution of the mole vole (Table 3) include Dem, Bio7, Bio18, Bio2, Bio1, BSAT, Bio15, and TCARBON_EQ, with a cumulative contribution rate of 85.96%. Among these, Dem (Elevation) and Bio7 (Temperature annual range) exhibit the highest contribution rates, characterizing them as the most critical environmental determinants governing the potential geographical distribution of the mole vole.

### 3.3. Changes in Potential Suitable Habitat Area Under Future Climate Scenarios

**Figure 4:**
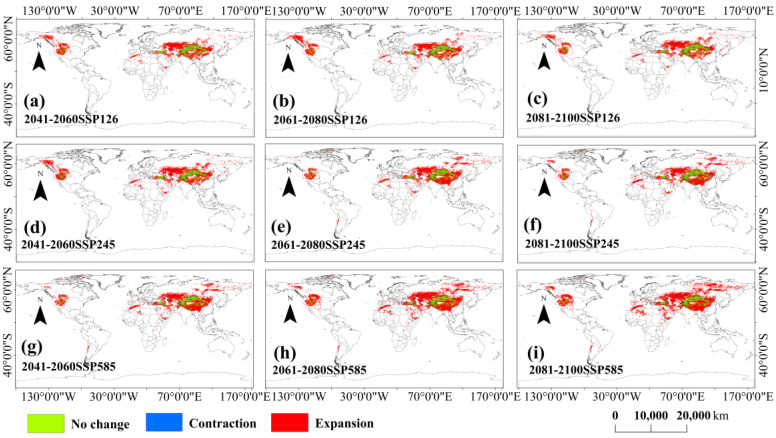
Changes in the potential suitable habitat for *E. tancrei* under different future climate scenarios compared to current conditions: (**a**) during the 2041–2060 period under the SSP1-2.6 scenario; (**b**) during the 2061–2080 period under the SSP1-2.6 scenario; (**c**) during the 2081–2100 period under the SSP1-2.6 scenario; (**d**) during the 2041–2060 period under the SSP2-4.5 scenario; (**e**) during the 2061–2080 period under the SSP2-4.5 scenario; (**f**) during the 2081–2100 period under the SSP2-4.5 scenario; (**g**) during the 2041–2060 period under the SSP5-8.5 scenario; (**h**) during the 2061–2080 period under the SSP5-8.5 scenario; and (**i**) during the 2081–2100 period under the SSP5-8.5 scenario.

As shown in Figure 4 and Table 4, the potential suitable habitats of *E. tancrei* exhibit trends of expansion, contraction, and stability relative to the current distribution across all three future periods and climate scenarios.

Specifically, under the SSP1-2.6, SSP2-4.5, and SSP5-8.5 scenarios, for the 2041–2060 period, the habitat expansion areas were projected to be 13.24 × 10^6^ km^2^, 15.06 × 10^6^ km^2^, and 15.97 × 10^6^ km^2^, respectively. Conversely, habitat contraction is limited to 3.83× 10^4^ km^2^, 2.79× 10^4^ km^2^, and 3.99 × 10^4^ km^2^, while the stable suitable habitat areas remain 3.25× 10^6^ km^2^, 3.26 × 10^6^ km^2^, and 3.25× 10^6^ km^2^, respectively. For the 2061–2080 period, the projected expansion increases to 13.81 × 10^6^ km^2^, 15.18 × 10^6^ km^2^, and 20.41 × 10^6^ km^2^, respectively. The contraction areas are 3.84 × 10^4^ km^2^, 4.50 × 10^4^ km^2^, and 3.68 × 10^4^ km^2^, with stable habitat areas of 3.25 × 10^6^ km^2^, 3.25 × 10^6^ km^2^, and 3.25 × 10^6^ km^2^, respectively. For the 2081–2100 period, the expansion trend further intensifies, reaching 14.36 × 10^6^ km^2^, 15.45 × 10^6^ km^2^, and 22.45 × 10^6^ km^2^, respectively. Contraction areas increase to 3.63 × 10^4^ km^2^, 4.39 × 10^4^ km^2^, and 2.89 × 10^4^ km^2^, while stable habitat areas slightly decrease to 3.25 × 10^6^ km^2^, 3.25 × 10^6^ km^2^, and 3.26 × 10^6^ km^2^, respectively.

The spatial patterns of habitat expansion, contraction, and persistence remain largely consistent across all scenarios and periods. Common expansion regions are primarily distributed in Eurasia (TUR, ESP, SAU, YEM, IRN, GEO, ARM, AZE, UKR, AFG, UZB, TJK, KAZ, CHN, MNG, RUS, etc.), North America (CAN, USA, etc.), South America (CHL, ARG, etc.), and Northern Africa (MAR, DZA, etc.). Common contraction regions are predominantly located in Eurasia (CHN, MNG, RUS, etc.) and North America (CAN, USA, etc.). Common persistence regions are mainly found in Eurasia (TUR, ARM, AZE, IRN, AFG, KAZ, KGZ, TJK, CHN, MNG, RUS, etc.), North America (USA, CAN, etc.), and Northern Africa (MAR, etc.) (Figure 4).

### 3.4. Centroid Shifts of Suitable Habitat Under Climate Change

**Figure 5:**
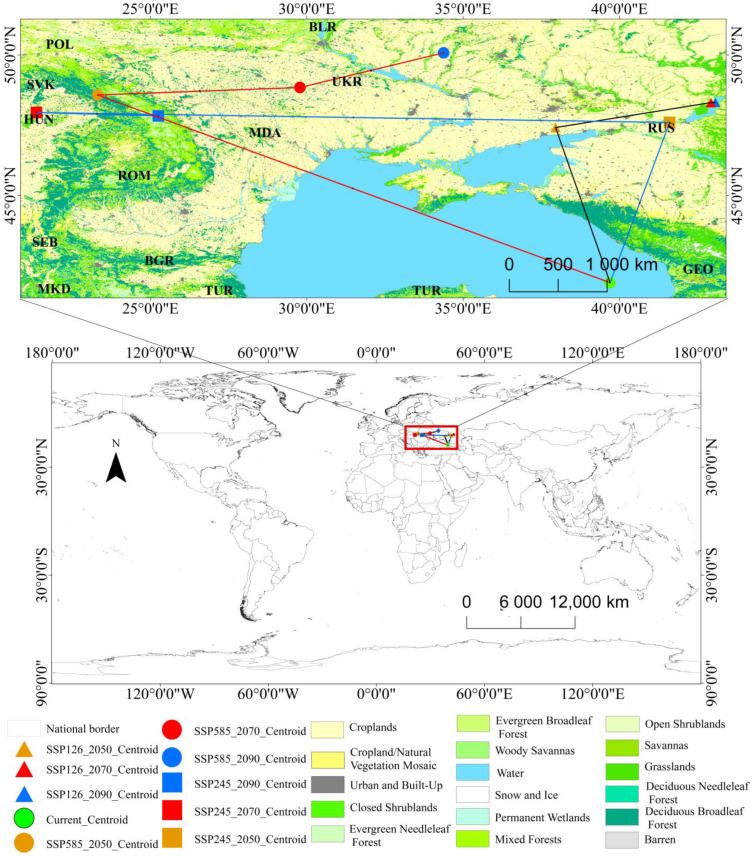
Migration trajectories of the habitat centroids for the mole vole (*E. tancrei*) across different climate scenarios. The black lines with arrows represent the linear migration distance and direction under the SSP126 scenario from the current baseline to the 2081–2100 period. The blue and red lines with arrows denote the corresponding centroid shifts under the SSP245 and SSP585 scenarios, respectively. Country Abbreviations: POL, Poland; SVK, Slovakia; HUN, Hungary; BGR, Bulgaria; BLR, Belarus; MDA, Moldova; UKR, Ukraine; SRB, Serbia; MKD, North Macedonia; ROM, Romania; TUR, Turkey; GEO, Georgia; RUS, Russian Federation.

As illustrated in Figure 5 and Table 5, the geometric centroid of the current suitable habitat for *E. tancrei* is located in the Black Sea region (39.700619° E, 42.110227° N), an area classified as water according to the global land-cover map. Therefore, changes in centroid elevation should be interpreted as geometric shifts in the spatial center of predicted suitable habitats rather than as actual altitudinal migration of the species.

Under the SSP126 scenario, the centroid is projected to shift to UKR (37.957504 E, 47.362569 N) in the 2041–2060 period and subsequently to RUS in 2061–2080 (42.923228 E, 48.256353 N) and 2081–2100 (43.067548 E, 48.261741 N). Relative to the preceding periods, the migration distances are 506 km, 14 km, and 487 km, respectively. In terms of vertical displacement, the mean elevation of the centroid increases by 2070 m in the 2041–2060s, followed by a decrease of 30 m in the 2061–2080s and a subsequent increase of 13 m in the 2081–2100s. The land-use type associated with the centroid locations is croplands during the first two periods (2041–2060s and 2061–2080s), shifting to grasslands in the 2081–2100s. Under the SSP245 scenario, the centroid is projected to be located in RUS during the 2041–2060 period (41.602428 E, 47.544599 N), shifting to HUN in the intermediate 2061–2080 period (21.352231 E, 47.900665 N), and finally to ROM in the 2081–2100 period (25.252531 E, 47.778612 N). The migration distances relative to the previous periods are 1954 km, 528 km, and 376 km, respectively. Elevation increases by 1985 m in the first period, followed by further increases of 80 m and 912 m in the second and third periods, respectively. The land-use type associated with the centroid locations is croplands during the first two periods (2041–2060s and 2061–2080s), shifting to woody savannas in the 2081–2100s.

Under the SSP585 scenario, the centroid is projected to shift to POL (17.412976 E, 51.77965 N) in 2041–2060, DEU (14.084049 E, 52.335388 N) in 2061–2080, and POL (19.825168 E, 50.315692 N) in 2081–2100. The migration distances relative to the preceding periods are 1694 km, 458 km, and 613 km, respectively. Elevation increases by 2822 m in the first period, decreases by 615 m in the second period, and then drops by 105 m in the final period. The land-use types associated with the centroid locations transition from deciduous broadleaf forests (savanna margins) to croplands, and remain as croplands in the final period.

Overall Directional Trends: Under the SSP245 and SSP585 scenarios, the centroid migrates northwestward relative to the current period, whereas under the SSP126 scenario, it migrates northeastward.

## 4. Discussion

### 4.1. Model Accuracy Evaluation and Dominant Environmental Factors

Climatic factors such as temperature and precipitation are primary determinants of the geographical distribution of wildlife species [40]. In this study, we developed a predictive distribution model for the mole vole (*E. tancrei*) characterized by high accuracy and reliability [41]. When constructing species distribution models (SDMs), the selection of environmental variables and sample sizes significantly influences the predictive outcomes [42]. Studies that have relied solely on climatic factors to predict species distributions [43,44] have inherent limitations [45]. To enhance the precision of our predictions for the potential suitable habitats of the mole vole, we integrated 38 environmental variables, including 19 bioclimatic factors, elevation, slope, aspect, and 16 soil factors. Following redundancy elimination through contribution analysis, Pearson correlation, and Variance Inflation Factor (VIF) analysis, 15 variables were initially screened. Subsequently, through individual model evaluation and calculation of the average percentage contribution of each variable, we identified the key factors driving the species’ distribution. Notably, elevation (Dem) and temperature annual range (Bio7) exerted the most significant influence on habitat selection.

For subterranean rodents, maintaining relatively stable microclimatic conditions within burrow systems is vital. These burrow environments not only support essential behaviors such as foraging and reproduction, but also provide a buffer against predators and adverse fluctuations in the external environment [46]. The internal temperature of these burrows is regulated by surface temperatures, with the degree of influence being contingent upon burrow depth and soil properties [47]. Therefore, our findings suggest that annual mean temperature likely regulates internal burrow temperatures by influencing surface thermal dynamics, thereby dictating the distribution of the mole vole. Furthermore, the mole vole’s diet primarily consists of plant roots and tubers [20]. Soil water resources are a fundamental component of soil ecosystems, and they are essential for the survival of vegetation [48]. The availability of food resources and habitat vegetation cover are critical factors in habitat selection for rodents, and population fluctuations are directly linked to food availability [49]. Consequently, soil available water capacity was identified as a critical environmental determinant for the survival and persistence of the mole vole.

Climatic factors, such as temperature and precipitation, are primary determinants of the geographical distribution of wildlife species. In this study, we developed an ensemble species distribution model for the mole vole (*E. tancrei*) with high predictive performance. The final ensemble model achieved high values of both TSS and AUC, indicating excellent discrimination capacity and reliability for projecting the potential suitable habitats of *E. tancrei*. When constructing species distribution models (SDMs), the selection of ecologically meaningful environmental variables and the quality of occurrence records can substantially influence model performance and ecological interpretation. Therefore, to improve the robustness of habitat suitability prediction, we integrated climatic, topographic, and soil variables, and further reduced multicollinearity and redundancy through contribution analysis, Pearson correlation analysis, and variance inflation factor screening.

Among the 15 environmental variables retained in the final ensemble model, elevation (Dem) and temperature annual range (Bio7) showed the highest contributions, indicating that the potential distribution of *E. tancrei* is primarily constrained by topographic gradients and seasonal thermal variability rather than by mean climatic conditions alone. This result is ecologically reasonable because elevation is not a single direct resource, but an integrative variable that reflects covarying changes in temperature, atmospheric pressure, soil development, vegetation type, moisture availability, snow cover, and land-use patterns. Previous ecological studies have emphasized that elevational gradients provide strong natural gradients for examining biological responses to environmental variation, especially temperature-related constraints [50,51]. In species distribution modelling, the ecological relevance and spatial scale of predictor variables strongly affect model interpretation and projection reliability; therefore, the high contribution of Dem suggests that the distribution of *E. tancrei* is closely linked to complex mountain–steppe and piedmont environmental gradients rather than to simple horizontal climatic zonation [52,53,54].

This interpretation is also consistent with the field occurrence data used in this study. Our field records covered multiple grassland types, including temperate desert, temperate desert steppe, temperate steppe, temperate meadow steppe, alpine steppe, and artificial grassland, with an elevation range of 649–3218 m. Such a broad elevational span suggests that *E. tancrei* can occupy heterogeneous habitats, but its suitability is likely highest within specific topographic belts where thermal conditions, soil properties, vegetation structure, and belowground food resources jointly meet the ecological requirements of this semi-subterranean rodent. Similar patterns have been reported for small mammals, whose diversity and distribution along elevational gradients are often shaped by climatic, spatial, and habitat-related mechanisms rather than by elevation per se [55,56].

Bio7, defined as the difference between the maximum temperature of the warmest month and the minimum temperature of the coldest month, represents annual thermal amplitude and reflects the intensity of seasonal temperature fluctuation [57]. The high contribution of Bio7 is particularly relevant for *E. tancrei*, because although this species is widely recorded in deserts, semi-deserts, and arid steppes of Central Asia, our field records further show that it also occurs in temperate steppes, meadow steppes, alpine steppes, and artificial grasslands across a broad elevational gradient. Such habitat heterogeneity indicates that *E. tancrei* is not restricted to a single arid habitat type, but occupies landscapes with pronounced seasonal thermal contrasts. Therefore, Bio7 may capture the annual thermal amplitude that constrains overwintering survival, reproductive timing, burrow microclimate, and shallow foraging activity. For semi-subterranean rodents, burrow systems can buffer short-term surface temperature variation, but burrow microclimate is still influenced by regional climate, soil thermal properties, burrow depth, soil texture, rainfall, and seasonal temperature regimes [58,59,60,61]. Therefore, Bio7 may affect habitat suitability by regulating the energetic costs of thermoregulation, overwintering survival, reproductive timing, and the use of shallow foraging tunnels.

The dominance of Dem and Bio7 further suggests that the potential distribution of *E. tancrei* is controlled by the interaction between topographic filtering and seasonal thermal stress. Elevation determines the broad environmental template by shaping regional temperature, moisture, vegetation, soil development, and landform conditions, whereas Bio7 determines whether the annual thermal regime remains within the species’ physiological and ecological tolerance range. Although Bio18, Bio2, Bio1, and soil-related variables such as BSAT, AWC, CLAY, and TCARBON_EQ also contributed to habitat suitability, these factors should be interpreted as secondary but ecologically meaningful constraints. They may influence warm-season vegetation productivity, soil moisture retention, burrow stability, and the availability of roots and tubers. Overall, our results indicate that the potential geographical distribution of *E. tancrei* is shaped mainly by elevation-related habitat heterogeneity and annual temperature amplitude, with precipitation and soil properties acting as local modifiers of habitat suitability.

### 4.2. Analysis of Habitat Suitability and Distribution Dynamics

The occurrence records of the mole vole (*E. tancrei*) documented in our field surveys encompass a wide range of grassland types, including temperate desert, temperate desert steppe, temperate steppe, temperate meadow steppe, alpine steppe, and artificial grassland, with an elevational span ranging from 649 to 3218 m. Compared to other common wild rodents, *E. tancrei* exhibits a broader distribution across highly complex and diverse habitats. For instance, the great gerbil (*Rhombomys opimus*), another small rodent, primarily inhabits the Gobi Desert and saline shrublands dominated by *Haloxylon ammodendron* and *Kalidium foliatum* [62]. Similarly, the plateau zokor (*Eospalax baileyi*), a typical subterranean species, is an endemic rodent restricted to alpine meadows [63].

However, for species characterized by distant populations and extensive habitat diversity, it is highly probable that some areas harbor subspecies or cryptic species, as previously documented in relation to *Peromyscus* [64] and *Allactaga elater* [65]. Conversely, international studies have also confirmed that the mole vole occupies a vast array of habitats across its geographical range, exhibiting significant inter-population variations in demography and social structure [20,66]. This study adopted a spatial resolution of approximately 5 km for species distribution modeling, which is substantially coarser than the actual home range and microhabitat activity scale of *E. tancrei*. Such a relatively coarse spatial grain tends to smooth fine-scale environmental heterogeneity, leading to a broader predicted climatic suitability range and a certain overestimation of potential suitable habitats.

Despite the limitation of spatial scale mismatch, all environmental and climatic variables in this study were extracted strictly based on verified field occurrence points of the species, which effectively reduced sampling bias and ensured the overall reliability of modeling results. Future studies could integrate high-resolution microhabitat variables and adopt nested hierarchical modeling frameworks to further improve the accuracy and rationality of habitat prediction for small-range mammal species.

The intrinsic link between geographical isolation and species distribution remains a pivotal scientific question, as isolation often restricts species-range expansion [67]. Our model predicts that under both current and future climatic scenarios, highly suitable habitats for *E. tancrei* will be distributed across Central North America, Northern and Central Asia, and Southern Europe. While the predicted distributions in Eurasia and Southern Europe align closely with the current global occurrence of the species, no empirical records confirm its presence in Central North America (Southern Canada and the Northern United States) or Southern South America. This discrepancy suggests that while these regions meet the species’ threshold for ecological suitability, geographical barriers—most notably the Bering Strait—have likely impeded its natural dispersal. Similar constraints have been documented in regard to the distribution of *Bandicota indica* [32]. Furthermore, ecological suitability does not invariably guarantee species occurrence; for instance, invasive species often thrive in new environments with suitable habitats only after overcoming dispersal barriers via natural or anthropogenic pathways [68].

The current model predicted highly or moderately suitable habitats mainly in Eurasia, particularly in Turkey, China, Mongolia, Russia, and Kyrgyzstan, which is broadly consistent with the known distributional pattern of *E. tancrei*. However, some suitable areas were also projected in Central North America and other geographically isolated regions. These areas should be interpreted as environmentally suitable but not necessarily accessible habitats. Because *E. tancrei* has no confirmed natural populations in North America or South America, the predicted suitability in these regions likely reflects climatic similarity rather than realized distribution. Geographic barriers, dispersal limitation, historical biogeography, and the absence of natural colonization pathways may prevent the species from occupying these climatically suitable regions [69,70]. Therefore, our predictions represent potential ecological suitability rather than actual occurrence or inevitable range expansion.

Under future climate scenarios, the potential suitable habitats of *E. tancrei* showed simultaneous expansion, contraction, and persistence relative to the current distribution. Across all scenarios and periods, the expansion area was larger than the contraction area. This indicates that future climate change may create additional environmentally suitable space for *E. tancrei*. The SSP126, SSP245, and SSP585 scenarios represent different future climate pathways, ranging from low-emission mitigation to high-emission fossil-fuel development [71]. In this study, the expansion trend generally increased with emission intensity and time, suggesting that stronger climate change may alter the spatial configuration of suitable habitats for this species. Nevertheless, modelled habitat expansion should not be interpreted as direct evidence of realized population expansion, because actual range shifts also depend on dispersal capacity, habitat connectivity, available soil conditions, and population processes.

Centroid shift analysis further revealed changes in the spatial center of suitable habitats under future climate scenarios. In our results, the centroid shifted northeastward under SSP126, whereas it shifted northwestward under SSP245 and SSP585 relative to the current period. This suggests that different emission pathways may lead to different spatial redistribution patterns of suitable habitats. Such directional changes are generally consistent with the broader expectation that climate warming can drive species distributions toward higher latitudes or higher elevations [72,73]. However, the centroid should be interpreted as a geometric summary of predicted suitable habitat patches, not as an actual occurrence locality. The current centroid falling in the Black Sea region reflects the spatial balance among discontinuous suitable patches, rather than a real habitat of *E. tancrei*. Therefore, centroid migration indicates the overall direction of habitat suitability redistribution, but it should not be regarded as direct evidence of rapid long-distance migration.

The land-cover types associated with future centroids also provide useful ecological implications, but they should be interpreted cautiously. In our projections, several future centroid locations were associated with croplands, grasslands, woody savannas, or forest–savanna transition zones. Because *E. tancrei* mainly feeds on belowground plant organs such as roots and tubers [20], overlap between suitable climatic conditions and agricultural or grassland landscapes may increase potential rodent damage risk. Rodents are widely recognized as important agricultural pests that can damage crops, stored products, and grassland resources [74]. However, this does not mean that *E. tancrei* will necessarily colonize distant agricultural regions such as Europe or North America. A more cautious interpretation is that agricultural–pastoral ecotones within or near the known and realistically accessible range of the species may face increased potential risk under future climate change. Therefore, monitoring and early-warning management should prioritize Central Asia, northwestern China, Mongolia, southern Russia, and adjacent agricultural–pastoral transition zones.

Finally, several uncertainties should be acknowledged. First, although occurrence records were carefully filtered and spatially rarefied, public biodiversity databases may still contain taxonomic misidentifications or georeferencing errors, which are particularly important for morphologically similar semi-subterranean rodents. Second, soil and topographic variables were treated as static predictors in future projections because spatially continuous future soil datasets are unavailable. In reality, soil properties may change under desertification, erosion, agricultural expansion, and land-use conversion. Third, the present model did not explicitly incorporate dispersal barriers, interspecific interactions, dynamic land-cover change, or demographic processes. Future studies should combine verified field records, genetic or morphological identification, high-resolution microhabitat data, dynamic land-cover projections, and dispersal constraints to improve the biological realism of habitat suitability and range-shift predictions.

## 5. Conclusions

(1) The potential distribution of *E. tancrei* is mainly driven by topographic and climatic factors. Among the 15 environmental variables retained in the final model, elevation (Dem) and temperature annual range (Bio7) showed the highest contributions, indicating that habitat suitability is primarily constrained by elevation-related habitat heterogeneity and annual thermal variation. Precipitation of the warmest quarter (Bio18), mean diurnal range (Bio2), annual mean temperature (Bio1), base saturation (BSAT), precipitation seasonality (Bio15), calcium carbonate content (TCARBON_EQ), and available water capacity (AWC) also contributed to the model, but they acted as secondary environmental constraints.
(2) Under current climate conditions, the highly suitable habitats for *E. tancrei* are mainly concentrated in Eurasia, particularly in Turkey, Kyrgyzstan, China, Mongolia, and Russia. Moderately suitable habitats are mainly distributed in Turkey, Iran, Kyrgyzstan, Tajikistan, Russia, China, Mongolia, and Kazakhstan.
(3) Under future climate scenarios, the potential suitable habitats of *E. tancrei* are projected to show simultaneous expansion, contraction, and persistence relative to the current distribution. Across all future periods and SSP scenarios, the expansion area is larger than the contraction area, suggesting that climate change may increase the extent of environmentally suitable habitats for this species. The expansion trend is generally stronger under higher-emission scenarios, especially under SSP585 in the late 21st century.
(4) The centroid of suitable habitats shows scenario-dependent shifts under future climate change. Relative to the current period, the centroid shifts northeastward under SSP126, whereas it shifts northwestward under SSP245 and SSP585.

## Figures and Tables

**Table 1:** Environmental variables used for model construction using the biomod2 package (version 4.2-6-2).

Environmental Variable	Description	Unit
AWC	Available water capacity	mm
BSAT	Base saturation	%
Bio1	Annual mean temperature	°C
Bio15	Precipitation seasonality	%
Dem	Elevation	m
Bio7	Temperature annual range	°C
Bio2	Mean diurnal range	°C
GYPSUM	Gypsum content	%
CEC_SOIL	Cation Exchange Capacity of Soil	cmol (+)/kg
TEXTURE_SOTER	Texture class (SOTER conventions)	
Slope	Slope	%
Bio18	Precipitation of the Warmest Quarter	mm
TCARBON_EQ	Calcium carbonate	%
Aspect	Aspect	(°)
CLAY	Clay content	%

**Table 2:** TSS and AUC values for each model.

Model	TSS	AUC
CTA	0.848	0.956
FDA	0.816	0.964
GAM	0.834	0.972
GBM	0.89	0.987
GLM	0.939	0.995
MARS	0.888	0.978
MAXENT	0.887	0.957
RF	1	1
RFd	0.989	0.999
SRE	0.589	0.794
XGBOOST	0.99	0.996
EMmean	0.982	0.999

Note: The model selection criteria were a TSS ≥ 0.7 and an AUC ≥ 0.8. Only individual models meeting these thresholds were retained and incorporated into subsequent analyses; models that failed to meet these criteria were excluded.

**Table 3:** Contribution rates of all environmental variables under the current climate conditions.

Environmental Variable	Percent Contribution (%)
Dem	24.7
Bio7	23.2
Bio18	9.34
Bio2	9.04
Bio1	8.51
BSAT	4.02
Bio15	3.63
TCARBON_EQ	3.52
AWC	3.10
CLAY	2.74
Slope	1.99
CEC_SOIL	1.80
GYPSUM	1.71
TEXTURE_SOTER	1.62
Aspect	1.12

**Table 4:** Changes in the suitable habitat area for *E. tancrei* under different climate scenarios relative to the current period.

Period	Climate Scenario	Expansion in Area (km^2^)	Contraction in Area (km^2^)	Area of Stable Suitable Habitat (km^2^)
2041–2060s	SSP126	13.24 × 10^6^	3.84 × 10^4^	3.25 × 10^6^
	SSP245	15.06 × 10^6^	2.79 × 10^4^	3.26 × 10^6^
	SSP585	15.97 × 10^6^	3.99 × 10^4^	3.25 × 10^6^
2061–2080s	SSP126	13.81 × 10^6^	3.84 × 10^4^	3.25 × 10^6^
	SSP245	15.18 × 10^6^	4.50 × 10^4^	3.25 × 10^6^
	SSP585	20.41 × 10^6^	3.68 × 10^4^	3.25 × 10^6^
2081–2100s	SSP126	14.36 × 10^6^	3.63 × 10^4^	3.25 × 10^6^
	SSP245	15.45 × 10^6^	4.39 × 10^4^	3.25 × 10^6^
	SSP585	22.45 × 10^6^	2.89 × 10^4^	3.26 × 10^6^

**Table 5:** Coordinates and elevation of the suitable habitat centroids for *E. tancrei* under different climate scenarios.

Climate Scenario	Period	Coordinates of the Centroid	Elevation (m)
	Current period	39.700619 E, 42.110227 N	−1975
SSP126	2041–2060s	37.957504 E, 47.362569 N	95
	2061–2080s	42.923228 E, 48.256353 N	65
	2081–2100s	43.067548 E, 48.261741 N	78
SSP245	2041–2060s	41.602428 E, 47.544599 N	10
	2061–2080s	21.352231 E, 47.900665 N	90
	2081–2100s	25.252531 E, 47.778612 N	1002
SSP585	2041–2060s	23.310574 E, 48.53278 N	847
	2061–2080s	29.783008 E, 48.804057 N	232
	2081–2100s	34.377215 E, 50.084489 N	127

## Data Availability

Publicly available datasets were analyzed in this study. This data can be found here: current and future bioclimatic data, alongside the Digital Elevation Model (DEM), were obtained from WorldClim (https://www.worldclim.org (accessed on 20 December 2024)); soil data (Version 2.0) were downloaded from the Harmonized World Soil Database (HWSD) via the FAO portal (https://gaez.fao.org/pages/hwsd (accessed on 21 December 2024)); a total of occurrence records (spanning from 1991 to 2025) were retrieved from the Global Biodiversity Information Facility (GBIF, https://www.gbif.org/citation-guidelines (accessed on 29 June 2026)); all data involved in this study are available upon request from the corresponding author. The occurrence data of *Ellobius tancrei* has been uploaded to Figshare, with the link as follows: https://doi.org/10.6084/m9.figshare.32989844.

## Supplementary Materials

The following supporting information can be downloaded at https://www.mdpi.com/article/10.3390/ani16142245/s1. Table S1: Model based solely on field occurrence data.
