# Supplementary material for: Impacts of Global Climate Change on Potential Habitat Distribution and Range Shifts of Semi-Subterranean Rodents
Source: Animals (Basel). 2026 Jul 20;16(14):2245. doi: 10.3390/ani16142245 (PMC13403980; doi:10.3390/ani16142245)
Supplement: Supplementary file 1 [file animals-16-02245-s001.zip › animals-4329685-supplementary.pdf]

Table S1 Model based solely on field occurrence data

Model	TSS	AUC
CTA	0.949	0.995
FDA	0.927	0.978
GAM	0.975	0.996
GBM	0.975	0.998
GLM	0.985	0.999
MARS	0.933	0.993
RF	1	1
RFd	0.995	0.999
SRE	0.493	0.746
XGBOOST	0.999	1
EMmean	0.995	0.999

Note: The model selection criteria were a TSS ≥ 0.7 and an AUC ≥ 0.8. Only individual models meeting these thresholds were retained and incorporated into subsequent analyses; models that failed to meet these criteria were excluded.
